# Maize Combined Insect Resistance Genomic Regions and Their Co-localization With Cell Wall Constituents Revealed by Tissue-Specific QTL Meta-Analyses

**DOI:** 10.3389/fpls.2018.00895

**Published:** 2018-07-05

**Authors:** Arfang Badji, Michael Otim, Lewis Machida, Thomas Odong, Daniel Bomet Kwemoi, Dennis Okii, Symphorien Agbahoungba, Natasha Mwila, Frank Kumi, Angele Ibanda, Stephen Mugo, Samuel Kyamanywa, Patrick Rubaihayo

**Affiliations:** ^1^Department of Agricultural Production, Makerere University, Kampala, Uganda; ^2^Cereals Program, National Crop Resource Research Institute, Kampala, Uganda; ^3^International Maize and Wheat Improvement Center, Nairobi, Kenya

**Keywords:** maize, stem borers, storage pests, cell wall constituents, tissue-specific meta-QTL, multiple-insect resistance, marker-assisted selection

## Abstract

Combinatorial insect attacks on maize leaves, stems, and kernels cause significant yield losses and mycotoxin contaminations. Several small effect quantitative trait loci (QTL) control maize resistance to stem borers and storage pests and are correlated with secondary metabolites. However, efficient use of QTL in molecular breeding requires a synthesis of the available resistance information. In this study, separate meta-analyses of QTL of maize response to stem borers and storage pests feeding on leaves, stems, and kernels along with maize cell wall constituents discovered in these tissues generated 24 leaf (LIR), 42 stem (SIR), and 20 kernel (KIR) insect resistance meta-QTL (MQTL) of a diverse genetic and geographical background. Most of these MQTL involved resistance to several insect species, therefore, generating a significant interest for multiple-insect resistance breeding. Some of the LIR MQTL such as LIR4, 17, and 22 involve resistance to European corn borer, sugarcane borer, and southwestern corn borer. Eleven out of the 42 SIR MQTL related to resistance to European corn borer and Mediterranean corn borer. There KIR MQTL, KIR3, 15, and 16 combined resistance to kernel damage by the maize weevil and the Mediterranean corn borer and could be used in breeding to reduce insect-related post-harvest grain yield loss and field to storage mycotoxin contamination. This meta-analysis corroborates the significant role played by cell wall constituents in maize resistance to insect since the majority of the MQTL contain QTL for members of the hydroxycinnamates group such as p-coumaric acid, ferulic acid, and other diferulates and derivates, and fiber components such as acid detergent fiber, neutral detergent fiber, and lignin. Stem insect resistance MQTL display several co-localization between fiber and hydroxycinnamate components corroborating the hypothesis of cross-linking between these components that provide mechanical resistance to insect attacks. Our results highlight the existence of combined-insect resistance genomic regions in maize and set the basis of multiple-pests resistance breeding.

## Introduction

Maize (*Zea mays*) is one of the most essential cultivated food crops worldwide (Kanyamasoro et al., 2012). However, maize production is adversely affected by insect pests (Meihls et al., 2012). Stem borers (SB), and field-to-storage pests are the most devastating on cultivated maize (Demissie et al., 2008; Shiferaw et al., 2011). In Africa, the spotted stem borer (SSB) (*Chilo partellus*), the African maize stem borer (AMSB) (*Busseola fusca*), the African pink stem borer (*Sesamia calamistis*), and the African sugarcane borer (*Eldana saccharina*) are the SB species attacking maize (Stevens, 2008). Regarding storage pests (SP), the maize weevil (MW) (*Sitophilus zeamais*), and the Larger grain borer (LGB) (*Prostephanus truncatus*) are the most challenging to maize storability (Mwololo et al., 2012). In East Africa including Uganda, stem borers, SSB, and AMSB, and storage pests, MW, and LGB are the most abundant insect pest species with SSB being the most competitive species that can displace any indigenous field insect pest within not more than 2 years (Samayoa et al., 2015b). These insect pests account for losses ranging from 20 to 90% starting from the field through to the grain storage period (Nyukuri et al., 2014), with both SB and SP being responsible for contamination of grain with mycotoxins like aflatoxin and fumonisins (Cao et al., 2014). These substantial yield losses and health concerns prompted the use of several control methods aimed at inhibiting insect pest attacks on both maize plants and grains. Chemical control methods (Sylvain et al., 2015) and transgenic resistance conferred by *Bacillus thuringiensis* (*Bt*) have limitations such as applicability (Munyiri et al., 2015) and acceptability, and some of the critical pests can develop resistance to both or either insecticides or *Bt* proteins (Campagne et al., 2013). Besides, environmental factors are a crucial element in plant defensive mechanisms (Stam et al., 2014), and climate change is predicted to negatively impact on plant-insect interaction leading to less fitness of plants coupled with aggravated yield losses (Kissoudis et al., 2014).

Host plant resistance (HPR) is the best integrated-pest management option (García-lara et al., 2010; Murenga et al., 2016) since in its highest level it can reduce plant yield loss from insect pest attacks without the use of controversial methods such as insecticides or transgenic resistance. HPR is the inherent resistance of a plant to biotic stresses conferred by its genetic makeup. Thus to achieve good HPR, the genetic basis of the resistance needs to be understood. Past studies established the polygenic nature of maize resistance to insect pests in general, and SB and SP resistance, in particular, were found to have low to moderate heritability values (Bergvinson, 1999; Kim and Kossou, 2003; Sandoya et al., 2010; Barros et al., 2011). Both significant general and specific combining abilities (GCA, SCA) govern maize resistance to SB (Udaykumar et al., 2013) and SP (Kim and Kossou, 2003; García-lara et al., 2009) implying the importance of both additive and non-additive gene actions coupled with a significant influence of genotype by environment interactions (André et al., 2003; Sandoya et al., 2010; Barros et al., 2011). The development of insect resistant maize lines through conventional means received considerable efforts. Over the years, the International Maize and Wheat Improvement Center (CIMMYT) developed several Africa adapted maize populations resistant to multiple SB or SP (Tefera et al., 2016). However, no report of combined-resistance to both SB and SP is yet available. The nature of inheritance characterizing maize resistance to SB ad SP makes conventional breeding for resistance a challenging task (Murenga et al., 2016). An alternative to this challenge is the use of DNA molecular marker-assisted selection (MAS) to fix resistance genes in susceptible backgrounds of agronomic interest (André et al., 2003).

Therefore, toward the application of MAS in maize breeding, several studies investigated the genomic regions controlling maize resistance to insect pests using family-based quantitative trait loci (QTL) analyses. These studies concerned SP species such as MW (García-lara et al., 2009; Mwololo, 2013; Castro-Álvarez et al., 2015) and LGB (Mwololo, 2013) and SB species such as the European corn borer (ECB) (Schön et al., 1993; Bohn et al., 2000; Cardinal et al., 2001, 2006; Jampatong et al., 2002; Krakowsky et al., 2002; Papst et al., 2004), the sugarcane borer (SCB) (Bohn et al., 1996, 1997; Groh et al., 1998), the Southwestern corn borer (SWCB) (Bohn et al., 1997; Groh et al., 1998; Khairallah et al., 1998; Brooks et al., 2005, 2007), the Mediterranean corn borer (MCB) (Ordas et al., 2009, 2010; Samayoa et al., 2014, 2015a; Jiménez-Galindo et al., 2017), and SSB and AMSB (Munyiri and Mugo, 2017). However, due to the polygenic nature of insect resistance in maize, these studies resulted in the discovery of a plethora of QTL with mainly low phenotypic effects. Furthermore, for MAS to be more efficient than phenotypic selection, several requirements are bound to the used QTL. These criteria pertain to the precision of the positions and the genotypic effects of the QTL, and the QTL explaining a sufficient portion of the genotypic variance, yet most of the QTL detected fall short of these prerequisites (Utz et al., 2000; Chen et al., 2017). Besides, some QTL go undetected due to their small size in the populations under consideration (Bohn et al., 1997). Therefore, a comparative analysis of the genomic regions responsible for maize resistance to insects of similar feeding behaviors across studies could help to better understand the genetics of maize resistance to insects through the reduction of the plethora of reported QTL, and also, to propose the most valuable QTLs to perform MAS (Jiang, 2013).

On the other hand, previous studies explored the biochemical basis of maize resistance to insects including SB and SP. Meihls et al. (2013) reported the concentration of insect resistance-related QTL in some bins such as at the top of chromosome 1, the bottom of chromosome 2, and on chromosome 7 and that only 10% of maize bins are known to be involved in some insect resistance (Meihls et al., 2012). Moreover, stem boring resistance QTL co-localize with several QTL of defense chemicals in 51 bins (Meihls et al., 2012). Cell wall components (CWC), especially fiber and hydroxycinnamates provide maize resistance to feeding by several stem borers (Cardinal and Lee, 2005; Krakowsky et al., 2007; Santiago et al., 2016). Fiber components such as acid detergent fiber (ADF), neutral detergent fiber (NDF), and lignin, and hydroxycinnamates such a *p*-coumaric acid (*p*-CA), ferulic acid (FA), and diferulic acid (DiFA), which are byproducts of the phenylpropanoids pathway, are involved in maize resistance to ECB, SCB, SWCB, and MCB both in leaves and stems (Santiago et al., 2013, 2017). Besides, the hydroxycinnamates and several other cell wall bound constituents are also associated with maize kernels resistance to MW (García-lara et al., 2010; Castro-Álvarez et al., 2015). Co-localizations of QTL for insect resistance and CWC have often been reported (Cardinal and Lee, 2005; Krakowsky et al., 2007; Santiago et al., 2016).

However, the accumulations and involvement of biochemical compounds in plants resistance to insects is complex and varies highly from one genotype to another, one plant tissue to another, and even the same tissue, from one developmental stage to another (Santiago et al., 2013). Therefore, for each maize tissue, a Meta-QTL analysis of QTL identified for maize resistance to insects and QTL for CWC would allow a better understanding of the genetic and biochemical basis of resistance. Meta-analysis of QTL is a means for refining the positions of QTL on a consensus map developed from the integration of individual maps or their projection on a reference map to accurately detect consensus QTL across studies, genetic backgrounds, and environments (Sosnoswki and Joets, 2012). It generates useful information for molecular breeding and cloning and presents an efficient way of investigating genetic correlation among traits (Wang et al., 2016). Furthermore, QTL meta-analysis helps in mitigating some of the weaknesses of individual QTL that hinder their efficiency in MAS. In the context of maize, this approach holds promise for the identification of MQTL across germplasms of various genetic and geographical backgrounds since the pan-genome theory implies that virtually all the lines share a significant portion of the genomic regions containing almost all the genes (Morgante et al., 2007). On that note, the ultimate goal of this study was to conduct a comparative mapping of maize resistance to SB and SP along with CWC QTL to identify tissue-specific resistance genomic regions for use in multiple insect pest resistance molecular breeding. To achieve this goal, we used the IBM2 2008 Neighbors (www.maizegdb.org) genetic linkage map which allows an increase in QTL resolution (Lee et al., 2002) as a reference first to conduct individual meta-analyses of QTL to identify tissue-specific meta-QTL (MQTL) for leaf, stem, and kernel resistance, and secondly, investigate combined resistance genomic regions.

## Materials and methods

### QTL experiment literature survey and data generation

We surveyed published QTL experiments on maize CWC and resistance to ECB, SCB, SWCB, MCB, MW, and on CWC on Google Scholar (https://scholar.google.com/), PubMed (https://www.ncbi.nlm.nih.gov/pubmed/), on the MaizeGDB Locus + QTL data center (http://www.maizegdb.org/data_center/locus), and the Gramene database QTL data center (http://archive.gramene.org/qtl/) (Table 1, Supplementary Material Presentation 1: Maps and QTL files). From all the experiments that were later considered for analysis, we either downloaded the maps from the MaizeGDB (http://www.maizegdb.org/data_center/locus) or, when not available, we generated them using the published maps (Supplementary Material Presentation 1: Maps and QTL files). When marker coordinates were unavailable, we used the Adobe reader distance measurement tool (https://helpx.adobe.com/acrobat/using/grids-guides-measurements-pdfs.html) to measure the intervals of the different markers on each chromosome for each map relative to the first marker positioned at the zero coordinate. Then, we used the provided scale to convert the distances from inches to centiMorgans (cM). However, due to non-availability of maps both from the online databases and the publications, we could not include some of the QTL experiments (Groh et al., 1998; Brooks et al., 2005, 2007). We also did not consider experiments for which the maps were built using single nucleotide polymorphism (SNP) markers (Orsini et al., 2012; Mwololo, 2013; Samayoa et al., 2015a; Munyiri and Mugo, 2017), because of a lack of shared markers with the other maps. Also, due to lack of similar markers with the consensus map, from the experiment by Méchin et al. (2001), we could only project chromosome 7 containing one QTL. For each of the maps, the information recorded included the population size and type, and the mapping function. Regarding the QTL data, parameters included were the QTL name, trait, LOD score of the QTL, the percentage of phenotypic variance explained by each QTL (R^2^), QTL most likely position and its confidence interval (CI) start and end. Some of the publications did not provide information on the R^2^ and the LOD scores (Khairallah et al., 1998; Fontaine et al., 2003; Papst et al., 2004; Samayoa et al., 2014). Where only the likelihood ratio statistics (LRS) was available (Papst et al., 2004), the LRS of each QTL was used to compute its LOD score using the formula: $LOD=\frac{LRS}{4.6}$ (Liu, 1997). Also, the individual LOD scores of the QTL were used to estimate *R*^2^ using the formula: *R*^2^ = 1−10^(−2*LOD*/*N*)^, where *N* is the population size (Van and McHale, 2017). The CIs of the QTL were transformed at 95% using the following formulas: $CI=\frac{k}{NxR^{2}}$, where *K* = *530* for F2 and F3 populations, and *K* = *163* for recombinant inbred lines (RILs) and inter-mated RILs (IRILs) (Darvasi and Soller, 1997).

**Table 1:** Summary of the genetic parameters from QTL mapping experiments.

**Parents**	**Map**	**Pop. size**	**Pop. type**	**No. QTL**	**Traits**	**Tissues**	**Authors**
B73xMo17 (Ref.)	IBM2 2008 neighbors	302	IRILs	373^**^			
B73HtxMo47	Jampatong_2002	244	F3	14	ECB	Leaves/Stems	Jampatong et al., 2002
B73xB52	Cardinal_2001	183	RILs	28	CWC	Stems	Cardinal et al., 2003
				9	ECB	Stems	Cardinal et al., 2001
	Schon_1993	300	F3	6	ECB	Stems	Schön et al., 1993
B73xDe811	Krakowsky_2002	147	F3	7	ECB	Stems	Krakowsky et al., 2004
	Krakowsky_2004	191	RILs	15	ECB	Stems	Krakowsky et al., 2004
				19	CWC	Stems	Krakowsky et al., 2005
				29	CWC	Leaves	Krakowsky et al., 2006
B73xMo17	Hazen_2003	302	IRILs	12	CWC	Kernels	Hazen et al., 2003
				3	MCB	Stems/Kernels	Ordas et al., 2009
CML131xCML67	Bohn_1996	190	F2	8	SCB	Leaves	Bohn et al., 1996
	Bohn_1997	171	F3	10	SCB	Leaves	Bohn et al., 1997
				6	SWCB	Leaves	Bohn et al., 1997
CML290 × Muneng-8128 C0 HC1-18-2-1-1	Garcia-Lara_2009	163	F2	39	CWC	Kernels	García-lara et al., 2010
				15	MW	Kernels	García-lara et al., 2009
D06xD408	Bohn_2000	230	F2	8	CWC	Stems	Bohn et al., 2000
				11	ECB	Stems	Papst et al., 2004
				6	ECB	Stems	Papst et al., 2004
EP125xPB130	Santiago_2016	285	F2	16	CWC	Stems	Santiago et al., 2016
				5	MCB	Stems/Kernels	Santiago et al., 2016
EP39xEP42	Ordas_2010	178	RILs	4	MCB	Stems	Ordas et al., 2010
EP42xA637	Samayoa_2014	144	RILs	4	MCB	Stems/Kernels	Samayoa et al., 2014
F271xF288	Courtial_2013	244	RILs	13	CWC	Stems	Courtial et al., 2014
				16	CWC	Stems	Fontaine et al., 2003
				15	CWC	Stems	Roussel et al., 2002
				10	CWC	Stems	Courtial et al., 2013
F2xIo	Mechin_2001	100	RILs	6	CWC	Stems	Méchin et al., 2001
F838xF286	Barriere_2008	242	RILs	21	CWC	Stems	Barriere et al., 2008
Fl1xF2	Riboulet_2008	140	RILs	4	CWC	Stems	Riboulet et al., 2008
Ki3xCML139	Khairallah_1998	472		6	SWCB	Leaves	Khairallah et al., 1998
Mo17xH99	Cardinal_2006_1	147	F2	5	ECB	Leaves	Cardinal et al., 2006
	Cardinal_2006_b	223	RILs	5	ECB	Leaves	Cardinal et al., 2006
P84xKilima	Castro-Alvarez_2015	100	RILs	7	MW	Kernels	Castro-Álvarez et al., 2015
Total number of QTL				382^*^			

*Pop. Size, number of lines composing the mapping population; Pop. Type, generation of the mapping population; F2 or F3, Population at the second or third generation of recombination; IRILs, Intermated recombinant inbred lines; RILs, Recombinant inbred lines*.

*Trait, CWC, Cell call constituents; ECB, European corn Borer; MCB, Mediterranean corn bore; MW, Maize weevil; SCB, Sugarcane borer; SWCB, Southwester corn Borer*.

* *Number of QTL from the considered studies*,

** *Number of QTL successfully projected and used in the analysis*.

The data from each experiment was checked to reduce overlapped QTL by considering only the one with the highest *R*^2^ to avoid bias in the meta-analysis by over-representing the same QTL (Truntzler et al., 2010; Sosnoswki and Joets, 2012). The QTL experiments included in this analysis encompassed population from temperate (USA and Europe), sub-tropical and tropical regions. Moreover, each of the populations used for QTL mapping of maize response to the MW had at least one of the parental lines containing African pedigree (García-lara et al., 2009; Castro-Álvarez et al., 2015).

### Map and QTL projection and consensus map construction

We loaded the different maps along with the QTL data (Supplementary Material Presentation 1: Maps and QTL files) in BioMercator 4.2 (Arcade et al., 2004) which integrates each QTL file with its corresponding map and check for common markers (at least 2) between each pair of maps included in the analysis to allow integration of the maps. However, maps displayed different sets of markers, and we could not compile them directly. Therefore, we used the high-density genetic linkage map of more than 1500 markers, the IBM2 2008 Neighbors (www.maizegdb.org, Supplementary Material Presentation 1: IBM2 2008 Neighbors) as a reference map and iteratively projected the experimental maps. The iterative map compilation tool implemented in BioMercator 4.2 allowed for the projection of QTL and loci from the individual genetic maps to the reference map. Common markers between homologous chromosomes were used to compute a specific ratio for each interval between pairs of shared markers, and a global ratio was implemented to project the remaining markers located above or below the first interval of shared markers and below the last interval of shared markers, respectively. In that process, the software automatically discarded inverted markers. Finally, BioMercartor used a homothetic function to project the QTL (Sosnoswki and Joets, 2012). We compiled the maps by starting with the maps showing the highest similarity with the reference map to avoid having some markers or QTL CIs spanning beyond the scope of the reference map and generating negative coordinates. When for a particular map, some markers or QTL still fell beyond the zero coordinate of the reference map, we discarded them from the original maps or QTL files, respectively (Jiang et al., 2016).

### Meta-analyses for leaf, stem, and kernel insect resistance

We conducted separate meta-analyses of damage resistance and CWC QTL discovered in each tissue attacked by SB and SP, namely leaves, stems, and kernels to identify tissue-specific MQTL we named leaf insect resistance (LIR), stem insect resistance (SIR) and kernel insect resistance (KIR). The QTL choice option of the BioMercator 4.2 (Arcade et al., 2004) was used in each of the analyses to select QTL reported for the tissue under consideration. For each meta-analysis, procedures followed two steps to determine the number of “real QTL” present on each chromosome from the QTL projected to the reference map by clustering all the QTL of each chromosome and refining the CIs of the QTL (Sosnoswki and Joets, 2012). In Meta-analysis step 1 of 2 (Veyrieras et al., 2007), QTL on each chromosome of the reference map were clustered assuming a normal distribution of QTL locations around their true locations and the reported CI and *R*^2^-values were used to derive their variances. The software used the Akaike information criterion (AIC), corrected Akaike information criterion (AICc and AIC3), Bayesian information criterion (BIC), and approximate weight of evidence (AWE) to determine the most likely MQTL models on each chromosome. The lowest value obtained from the five criteria became the number of “real QTL” on the chromosome and was used in Meta-analysis step 2 of 2 to generate MQTL with their positions, CIs, and percentage of the membership of the original QTL to each MQTL (Veyrieras et al., 2007).

### Further analysis of the MQTL

For each MQTL, we recorded the right and left flanking markers on the IBM2 2008 Neighbors (www.maizegdb.org) reference map and determined their physical positions on the maize B73 reference map version 2 (www.maizegdb.org) using the locus pair lookup tool (Andorf et al., 2010). When a flanking marker was not physically mapped, we used the next closest outer marker. We used the same procedure to determine the physical positions of the QTL from the insect resistance mapping experiments (Groh et al., 1998; Brooks et al., 2007) that we could not include in the meta-analyses. We then compared the physical positions of the QTL determined by their flanking markers with those of the meta-QTL to investigate possible co-localizations. For the experiments which used SNPs (Samayoa et al., 2015a,b; Jiménez-Galindo et al., 2017), we used the physical positions of the significant SNPs where available.

## Results

### QTL projection and consensus map summary

**Figure 1:**
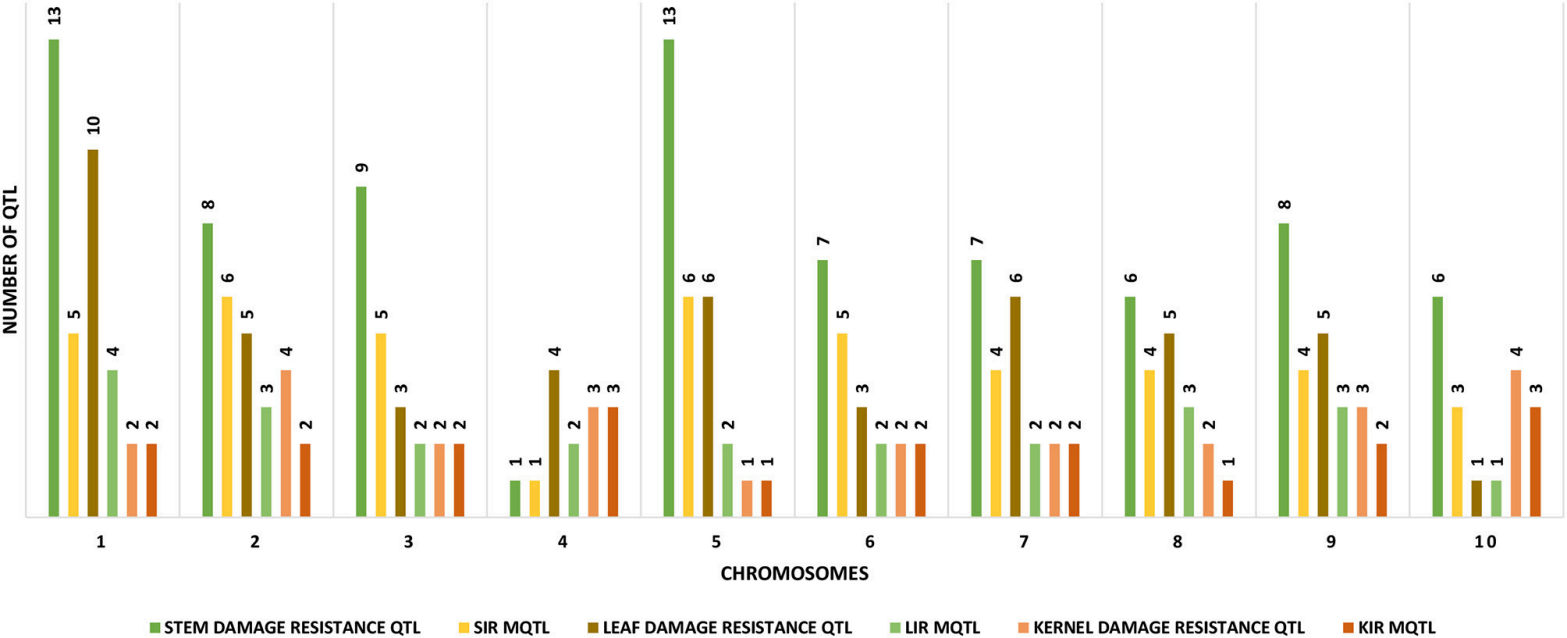
Distribution of the different tissue-specific MQTL LIR, Leaf insect resistance; SIR, Stem insect resistance; KIR, Kernel insect resistance, along with the original insect resistance QTL measured in each tissue.

We downloaded 302 QTL from 28 publications consisting of 32 experiments conducted on 21 populations derived from 17 crosses. Mapping populations comprised 10 recombinant inbred lines (RILs), one inter-mated recombinant inbred lines (IRILs), four F3s and six F2s developed from 19 crosses (Table 1). We successfully projected 383 QTL on the IBM2 2008 Neighbors reference map (www.maizegdb.org), of which, 152 were for insect resistance and 221 for CWC (Table 2). The individual maps projection to the reference map resulted in a consensus map of 16681 markers density (7980.637 cM) (Supplementary Material Presentation 1: map_CKM2_map and map_CKM2_QTL). The QTL spread on all the ten chromosomes with chromosome 1 having the highest numbers (57 QTL) and chromosome 8 with the lowest number (28) (Table 2, Figure 1).

**Table 2:** Distribution of the different QTL on the ten maize chromosomes.

**CHROMOSOME**	**1**	**2**	**3**	**4**	**5**	**6**	**7**	**8**	**9**	**10**	**TOTAL**
TOTAL NUMBER OF QTL	57	46	45	32	38	36	29	28	33	29	373
CWC	31	29	31	24	18	24	14	15	17	18	221
ECB	13	8	9	5	12	8	10	6	7	6	84
SCB	3	4	1	0	3	0	2	2	2	1	18
SWCB	3	0	1	0	3	1	1	1	2	0	12
MCB	5	2	1	0	1	1	0	3	3	0	16
MW	2	3	2	3	1	2	2	1	2	4	22
TOTAL INSECT RESISTANCE QTL											152
QTL IN STEMS	34	27	24	16	24	23	13	13	16	13	204
STEM DAMAGE RESISTANCE	13	8	9	1	13	7	7	6	8	6	78
QTL IN LEAVES	15	8	10	8	9	7	12	10	8	7	93
LEAF DAMAGE RESISTANCE	10	5	3	4	6	3	6	5	5	1	48
QTL IN KERNELS	8	11	11	8	5	6	4	5	9	9	76
KERNEL DAMAGE RESISTANCE	2	4	2	3	1	2	2	2	3	4	25

*CWC, Cell wall constituents; ECB, European corn borer; SCB, Sugarcane borer, SWCB, Southwestern corn borer; MCB, Mediterranean corn borer ; MW, maize weevil*.

### Summary of the leaf, stem, and kernel insect resistance MQTL

We identified 42 stem insect resistance (SIR), 42 leaf insect resistance (LIR), and 20 kernel insect resistance (KIR) MQTL (Tables 2, 3, Supplementary Material Presentation 1: MQTL summary). Each chromosome displays at least one of each of the tissue-specific resistance MQTL type (Figure 1) and shows a smaller number of the real QTL compared to the original QTL composition (Table 2). Chromosomes 1 contains the highest number of LIR MQTL, four MQTL, and chromosome 10 has the lowest, one MQTL. For SIR MQTL, chromosomes 2 and 5 have the highest, six MQTL each, and the lowest, one MQTL, on chromosome 4. Chromosomes 4 and 10 have each three KIR MQTL, and chromosomes 5 and 8 have each 1 KIR MQTL (Figure 1). The 95% confidence intervals (95% CI) for LIR MQTL vary from 7.71 cM for LIR10 to 140.45 cM for LIR7 with and an average of 48.04 cM. Meta-QTL for SIR have 95% CI varying from 4.73 for SIR11 to 191.02 cM for SIR16 and average at 26.30 cM. Regarding KIR MQTL, the 95% CI vary from 4.21 cM for KIR17 to 231.05.96 cM for KIR1 averaging at 24.42 (Table 3).

**Table 3:** Declared MQTL with their flanking markers and physical locations.

**Chr.Bin**	**MQTL**	**Start (cM)**	**Pos**.	**End (cM)**	**CI (95%)**	**Right and left flanking markers**	**Start and end physical positions (Mbp)**	**QTLs unmapped on the reference map#**
1.01	SIR1	48.06	57.39	65.53	18.53	bhlh140-fha1	4.74–5.75	
1.01–02	SIR2	100.5	112.72	122.33	22.94	php20689-ms26	10.07–14.50	
1.01–02	LIR1	80.82	134.13	185.67	106.27	idp4725-tidp6152	6.76–27.39	
1.04	SIR3	328.75	344.79	357.15	30.28	pza02114-umc2532	54.84–65.48	
1.04–06	KIR1	315.64	430.99	545.00	231.05	umc1452-uaz15	53.17–191.13	
1.05–06	LIR2	471.83	491.72	511.15	40.19	umc1395-bnlg1041	164.55–183.90	
1.06	SIR4	522.07	526.93	530.81	9.25	idp7408-csu256(hsp90)	183.77–188.13	
1.06–07	LIR3	601.80	610.06	617.09	16.21	gpm136-umc2237	198.10–200.72	
1.07	SIR5	667.33	674.08	678.25	11.30	idp4855-rz698a(ppy)	210.52–219.19	
1.09–10	KIR2	866.11	899.80	929.46	64.96	agrc362b-tlk1	262.07 −276.36	
1.10–11	LIR4	944.75	991.04	1034.31	91.32	idp2395-umc2514	279.67–291.89	SCB-LFD (Groh et al., 1998)
2.02	SIR6	78.03	90.12	102.14	24.45	umc1265-gpm914a	5.44–9.62	
2.02	LIR5	81.21	103.61	124.35	43.81	gpm470-tr1	5.44–12.13	
2.03–04	SIR7	244.25	250.80	255.67	12.10	bnlg381-ay103944	27.74–32.51	
2.04	SIR8	312.13	316.24	319.75	8.85	idp267-umc2079	49.81–57.11	
2.04	KIR3	311.50	321.20	330.53	19.96	idp8065-mmp89	48.40–61.92	
2.06	SIR9	376.21	385.08	392.70	17.65	umc1156-gpm738	154.52–186.56	
2.07–08	LIR6	456.15	475.69	492.94	36.96	idp6905-tidp3223	198.48–207.19	
2.07–08	LIR7	463.12	533.81	602.70	140.45	idp1657-pzb01013	198.48–225.61	
2.08	SIR10	479.62	494.74	507.53	29.16	idp660-umc116b	204.10–209.85	
2.08	KIR4	487.23	516.40	543.06	56.67	w3-tidp6470	204.43–215.01	
2.09	SIR11	594.45	597.43	598.25	4.73	idp8293-agrx825	222.39–224.60	
3.01–02	KIR5	7.39	38.80	68.20	62.24	gpm244-idp4717	1.59–4.87	
3.03	SIR12	106.82	112.94	116.68	11.26	csu728c-idp1482	8.11–10.08	
3.04	SIR13	141.57	248.13	251.43	11.42	csu1070-ay110151	67.63–110.72	
3.04	LIR8	262.11	273.87	283.48	22.95	csu851b-gpm835e	86.75–119.81	
3.05	SIR14	323.74	331.40	336.88	14.72	agrr179-umc1307	145.67–152.18	
3.05	SIR15	368.11	374.89	381.25	14.75	tidp2951-gpm397a	161.20–166.46	
3.06–08	KIR6	483.96	543.52	600.64	116.88	gpm513-idp8087	186.39–209.41	
3.08–09	LIR9	589.13	658.92	725.53	136.48	umc231-idp8203	206.61–218.85	
3.08–09	SIR16	616.25	712.10	806.88	191.02	wox9b-phot1	210.58–229.15	
4.01	LIR10	64.06	67.59	70.81	7.71	gpm521b -idp483	3.58–5.11	
4.03	SIR17	171.29	184.69	195.56	25.47	uaz180-gpm760b	17.42–18.62	
4.03–05	KIR7	191.52	217.31	242.77	52.59	idp7383-v17	17.93–40.45	
4.07–08	LIR11	425.52	436.68	445.75	21.57	pza03275-gpm151c	173.91–181.39	
4.08–09	KIR8	567.81	572.67	575.90	8.78	mdr1-rgpr3235b	202.51–216.53	
4.10	KIR9	655.48	674.68	693.24	38.70	umc1101- asg41	236.13–239.02	
5.02	SIR18	151.68	161.10	168.94	17.78	gpm359b-cdo542	8.46–12.29	
5.02	KIR10	184.35	204.82	225.05	41.61	idp8235-idp8641	12.29–20.00	
5.02	SIR19	247.24	253.12	258.34	11.77	idp5851-umc1151	29.90–42.38	MCB-TL (Samayoa et al., 2015a)
5.04	SIR20	313.22	325.25	335.41	22.41	umc1591-ay110906	135.81–162.66	
5.05	LIR12	397.07	415.03	431.60	35.24	idp4891-gpm922c	174.13–183.57	
5.05	SIR21	410.63	422.42	432.83	22.40	umc1687-tidp2809	180.15–185.72	
5.05	SIR22	478.46	483.18	485.86	7.97	phm532-bnlg1237	192.72–195.59	
5.06–07	LIR13	500.04	517.88	533.33	33.77	prr1-idp2459	201.26–205.36	
5.07–09	SIR23	644.31	654.03	662.81	18.68	ay110182-bnlg1885	205.44–217.01	
6.01	KIR11	68.88	85.47	101.90	34.02	po1-uaz169	9.41–75.24	
6.01	SIR24	79.70	91.43	102.47	23.06	mmp13-uaz169	13.92–75.24	
6.01	LIR14	79.13	94.08	107.59	28.96	mmp13-mmp10	13.92–70.95	
6.02–03	LIR15	148.58	163.80	177.04	28.72	mab26-php20856	93.49–104.54	
6.05	SIR25	315.95	325.82	335.30	20.09	ufg16-idp8048	144.27–148.36	
6.05	SIR26	369.46	372.96	375.15	5.96	isu1410i-nfy2	151.11–152.19	MCB-TL (Samayoa et al., 2015a)
6.06–07	KIR12	393.37	425.37	455.99	63.73	idp3915-npi9	153.37–162.29	
6.06–07	SIR27	422.97	436.49	449.16	27.01	lim151-phi299852	158.22–162.29	
6.07–08	SIR28	515.76	561.72	606.85	91.61	lhcb7-cdo202	164.90–END	
7.01–02	KIR13	120.32	135.87	151.12	31.29	idp1624-gpm804	10.47–17.36	
7.02	SIR29	177.35	213.57	249.20	72.94	idp8247-tidp3642	40.24–109.48	
7.02	LIR16	244.07	256.69	269.16	25.59	tidp8862-tidp2851	104.80–126.88	
7.03	SIR30	323.33	329.86	335.88	12.81	bnlg434-brd103	132.53–138.76	
7.03	KIR14	313.64	331.10	347.85	35.04	idp8017-gpm472	131.98–141.62	
7.03	LIR17	322.76	348.96	372.94	50.80	bnlg434-idd7	132.53–146.24	SWCB/SCB-LFD (Groh et al., 1998)
7.04	SIR31	486.99	498.92	509.80	23.01	gpm446b-cdo405	163.60–166.90	
7.04–05	SIR32	533.18	577.14	621.00	88.56	umc245-idp1466	168.36–170.99	
8.01–02	LIR18	104.41	133.28	161.35	57.60	gpm850a-pza02454	8.81–20.46	
8.03	SIR33	260.42	269.56	278.22	18.65	idp8347-pge2	94.99–101.39	
8.04–05	LIR19	307.29	321.21	333.39	26.16	gl18-cdo1081b	110.70–125.12	
8.04	SIR34	327.46	335.19	341.69	15.30	umc160b-rop7	119.11–122.95	
8.05–06	KIR15	396.86	401.78	405.17	8.49	umc1287-pza03182	141.95–160.44	
8.08	LIR20	521.44	544.70	567.71	47.02	tidp5576-cmu1	170.00–173.11	
8.08–09	SIR35	551.10	563.84	576.01	25.16	bnlg1056-dupssr14	171.74–175.44	
9.00–01	SIR36	9.55	23.71	35.81	27.35	rz144a-idp4166	2.78–7.33	
9.02	SIR37	108.03	118.40	128.32	21.24	umc256a-omt2	12.94–16.32	
9.02–03	LIR21	162.34	188.95	214.86	53.02	umc1037-w11	16.97–26.90	
9.03	KIR16	222.03	230.63	238.74	17.00	pza03469-idp2479	28.33–90.03	
9.03	SIR38	233.96	242.92	250.67	17.82	cdo319-tidp5661	26.96–99.16	
9.04	SIR39	294.24	297.95	300.21	5.99	csu263a-gpm622a	113.01–119.47	
9.04–05	LIR22	297.23	310.61	323.49	26.88	umc2398-lim458	114.05–133.59	SWCB/FAW-LDR (Brooks et al., 2007)
9.05–06	LIR23	378.40	407.06	435.69	57.64	idp708-rps22a	134.38–142.49	
9.06	KIR17	498.36	500.66	501.94	4.21	idp4802-gpm499	145.30–146.70	
10.03	SIR40	174.87	181.54	187.40	13.01	idp8241-cx1	42.25–64.26	MCB-SD (Jiménez-Galindo et al., 2017)
10.03	LIR24	202.33	211.79	219.67	17.75	ufg59-umc1938	66.71–83.67	
10.03–04	SIR41	233.64	240.50	246.17	13.06	idp1446-idp4425	87.12–97.76	
10.04	KIR18	260.14	267.21	273.63	13.57	odo1-bnlg2127	109.79–117.83	
10.05	SIR42	314.10	326.44	337.22	23.31	idp7650-gpm256	127.51–132.73	
10.07	KIR19	435.49	464.22	491.38	56.40	idp2467-rz17a	144.24–149.07	
10.07	KIR20	489.93	566.62	577.61	153.19	ren3-gpm835a	146.29–149.79	
**Genetic map coverage on the reference map**								
	QTL coverage (cM)^*^	**% of QTL genetic coverage ^#^**	MQTL coverage (cM)^*^	**% of MQTL genetic coverage ^#^**	**% of QTL to MQTL reduction @**			
KIR	2702.35	33.86	1110.38	13.91	58.91			
LIR	4024.24	50.43	1153.07	14.45	71.35			
SIR	5558.27	69.65	1104.83	13.84	80.12			

*Chr, Chromosome; LFM, Left flanking marker; RFM, Right flanking marker; KR, Kernel resistance; LFD, Leaf feeding damage; LDR, Leaf Damage Rating; SD, Stalk damage; ShR, Shank resistance; ST, Stalk tunneling; TL, Tunnel length; FAW, Fall armyworm; MCB, Mediterranean corn borer; SCB, Sugarcane borer; SWCB, Southwestern corn borer*.

# *Insect resistance QTL from studies not included in the meta-analysis but for which physical positions of their flanking markers were used to compare them with the positions of the MQTL*.

* *Genetic coverage of the QTL/MQTL in each tissue type on the reference map*.

# *Percentage of genetic coverage computed against the genetic length of the IBM reference map*.

@ *QTL to MQTL percentage of genetic coverage reduction achieved through meta-analysis for each tissue*.

Twenty-three SIR MQTL and 13 LIR MQTL involve at least two stem borer species with LIR4 and 22 combining three QTL for resistance to three different SB species (ECB, SCB, and SWCB). Regarding KIR MQTL, KIR3, 15, and 16 combine QTL for MW resistance with resistance to kernel damage by MCB (Table 4). Only KIR18 combines QTL for resistance to MW from both of the two studies conducted for response to MW (García-lara et al., 2009; Castro-Álvarez et al., 2015). Resistance to ECB is involved in most of the LIR, and SIR MQTL and several of these are specific to this insect. Most of the LIR, SIR, and KIR MQTL involve at least one CWC QTL except five LIR, five KIR, and four KIR QTL. Quantitative trait loci for fiber components and hydroxycinnamtes were co-evaluated only in stems, and the former were the only group measured in leaves and the latter were only measured in the kernels. Regarding SIR MQTL, fiber components and hydroxycinnamates are co-involved in 19 MQTL, while 14 MQTL contain only fiber components and three involve hydroxycinnamates alone (Table 4). Hydroxycinnamate QTL are involved in 14 KIR MQTL.

**Table 4:** Information of the QTL projected on each MQTL.

**MQTL**	**AUTHOR**	**TRAIT**	**QTL R^2^ (%)**
LIR1	Krakowsky et al., 2006	ADL	4.00
	Jampatong et al., 2002	ECB-LFD	4.70
LIR2	Cardinal et al., 2006	ECB-LBD	12.20
	Jampatong et al., 2002	ECB-LFD	11.80
	Bohn et al., 1997	SWCB-LDR	4.40
LIR3	Cardinal et al., 2003	SHADF	16.60
	Cardinal et al., 2003	SHNDF	24.90
	Bohn et al., 1997	SWCB-LDR	5.20
	Bohn et al., 1997	SCB-LDR	5.70
LIR4	Bohn et al., 1996	SCB-LFD	15.40
	Jampatong et al., 2002	ECB-LFD	9.00
	Cardinal et al., 2003	SHADL	5.40
	Bohn et al., 1997	SWCB-LDR	14.90
	Bohn et al., 1997	SCB-LDR	6.40
LIR5	Bohn et al., 1996	SCB-LFD	11.20
	Cardinal et al., 2003	SHADL	9.70
LIR6	Bohn et al., 1996	SCB-LDR	7.30
	Bohn et al., 1997	SCB-LFD	13.50
LIR7	Jampatong et al., 2002	ECB-LFD	5.80
	Bohn et al., 1997	SCB-LDR	20.20
LIR8	Bohn et al., 1997	SCB-LDR	3.80
	Krakowsky et al., 2006	ADF	8.00
	Krakowsky et al., 2006	NDF/ADF	4.00
	Cardinal et al., 2003	SHNDF	22.60
LIR9	Khairallah et al., 1998	SWCB-LFD	9.76
	Cardinal et al., 2006	ECB-LBD	5.00
	Krakowsky et al., 2006	NDF/ADF	5.00
LIR10	Jampatong et al., 2002	ECB-LFD	14.60
	Cardinal et al., 2006	ECB-LBD	42.90
	Cardinal et al., 2006	ECB-LBD	46.80
LIR11	Krakowsky et al., 2006	NDF/ADF	9.30
	Jampatong et al., 2002	ECB-LFD	16.00
LIR12	Bohn et al., 1996	SCB-LFD	10.10
	Cardinal et al., 2003	SHNDF	4.60
	Khairallah et al., 1998	SWCB-LFD	4.95
	Bohn et al., 1997	SCB-LDR	7.60
LIR13	Bohn et al., 1996	SCB-LFD	9.60
	Khairallah et al., 1998	SWCB-LFD	4.67
	Krakowsky et al., 2006	ADL	11.00
	Bohn et al., 1997	SWCB-LDR	6.30
LIR14	Krakowsky et al., 2006	NDF/ADF	11.00
	Cardinal et al., 2006	ECB-LBD	10.80
LIR15	Jampatong et al., 2002	ECB-LFD	15.00
	Khairallah et al., 1998	SWCB-LFD	4.93
LIR16	Cardinal et al., 2006	ECB-LBD	4.60
	Cardinal et al., 2003	SHADF	13.10
LIR17	Bohn et al., 1997	SWCB-LDR	1.60
	Cardinal et al., 2006	ECB-LBD	8.30
	Bohn et al., 1997	SCB-LDR	7.00
	Cardinal et al., 2006	ECB-LBD	3.90
	Bohn et al., 1996	SCB-LFD	10.90
LIR18	Khairallah et al., 1998	SWCB-LFD	5.83
LIR19	Bohn et al., 1996	SCB-LFD	10.40
	Krakowsky et al., 2006	ADL	4.00
	Krakowsky et al., 2006	ADF	10.00
	Cardinal et al., 2003	SHADL	8.60
	Cardinal et al., 2006	ECB-LBD	6.50
	Bohn et al., 1997	SCB-LDR	7.50
LIR20	Jampatong et al., 2002	ECB-LFD	4.40
	Krakowsky et al., 2006	ADL	16.00
	Krakowsky et al., 2006	NDF	11.00
LIR21	Krakowsky et al., 2006	NDF/ADF	11.00
	Bohn et al., 1996	SCB-LFD	8.70
LIR22	Cardinal et al., 2006	ECB-LBD	4.80
	Bohn et al., 1997	SWCB-LDR	8.10
	Bohn et al., 1997	SCB-LDR	30.80
	Cardinal et al., 2003	SHNDF	20.70
LIR23	Khairallah et al., 1998	SWCB-LFD	4.93
	Krakowsky et al., 2006	NDF	29.00
LIR24	Cardinal et al., 2003	SHADF	19.00
	Cardinal et al., 2003	SHNDF	16.30
	Krakowsky et al., 2006	ADL	9.00
	Krakowsky et al., 2006	ADL	8.00
	Krakowsky et al., 2006	ADL	6.00
	Bohn et al., 1997	SCB-LDR	8.40
KIR1	García-lara et al., 2009	MW-DI	1.00
KIR2	García-lara et al., 2010	CFP	8.80
	Castro-Álvarez et al., 2015	MW-AP	7.53
KIR3	Castro-Álvarez et al., 2015	MW-GWL	15.08
	García-lara et al., 2010	DFP	4.48
	García-lara et al., 2010	5,5'-DiFA	7.00
	Santiago et al., 2016	MCB-KR	3.29
KIR4	García-lara et al., 2010	trans-FA	1.08
	García-lara et al., 2009	MW-DI	3.30
	García-lara et al., 2010	8,5'-DiFA b	8.10
	García-lara et al., 2009	MW-GD	5.70
KIR5	Castro-Álvarez et al., 2015	MW-GWL	4.97
	García-lara et al., 2010	TPhA	5.69
KIR6	García-lara et al., 2009	MW-DI	6.20
	García-lara et al., 2010	trans-FA	4.08
KIR7	García-lara et al., 2010	p-CA	2.20
	García-lara et al., 2009	MW-GWL	5.40
KIR8	García-lara et al., 2010	8,5'-DiFA	3.40
	Hazen et al., 2003	Glc	6.60
	García-lara et al., 2010	8,5'-DiFA b	3.00
	Castro-Álvarez et al., 2015	MW-FP	6.97
KIR9	García-lara et al., 2009	MW-DI	8.00
KIR10	García-lara et al., 2010	8-O-4'-DiFA	1.20
	García-lara et al., 2009	MW-GD	7.30
KIR11	García-lara et al., 2010	TDiFA	8.10
	García-lara et al., 2009	MW-GWL	4.20
KIR12	García-lara et al., 2009	MW-GD	9.90
KIR13	García-lara et al., 2009	MW-GWL	9.90
KIR14	García-lara et al., 2009	MW-DI	3.70
	García-lara et al., 2010	trans-FA	8.03
KIR15	García-lara et al., 2010	HRGP-I	8.44
	Castro-Álvarez et al., 2015	MW-GWL	6.13
	Ordas et al., 2009	MCB-KD	5.50
KIR16	García-lara et al., 2010	DFP	5.78
	Santiago et al., 2016	MCB-KR	4.82
	García-lara et al., 2009	MW-AP	3.20
	García-lara et al., 2010	HRGP-I	16.80
KIR17	García-lara et al., 2009	MW-GWL	4.70
	García-lara et al., 2010	p-CA	11.60
	Hazen et al., 2003	Gal	12.40
KIR18	Hazen et al., 2003	Gal	5.91
	Castro-Álvarez et al., 2015	MW-FP	12.24
	García-lara et al., 2009	MW-AP	2.70
KIR19	García-lara et al., 2009	MW-GD	2.70
	García-lara et al., 2010	DFP	4.88
KIR20	Castro-Álvarez et al., 2015	MW-FP	11.17
SIR1	Barriere et al., 2008	ADL/NDF	6.40
	Papst et al., 2004	ECB-SDR	18.34
	Santiago et al., 2016	FA	5.60
	Courtial et al., 2013	KL/NDF	18.30
SIR2	Papst et al., 2004	ECB-SDR	3.88
	Krakowsky et al., 2002	ECB-ST	5.40
	Ordas et al., 2010	MCB-STL	11.60
SIR3	Krakowsky et al., 2002	ECB-ST	8.20
	Fontaine et al., 2003	Hcell	7.12
	Roussel et al., 2002	KL/NDF	11.80
	Santiago et al., 2016	DFAT	4.22
	Santiago et al., 2016	MCB-TL	3.29
	Santiago et al., 2016	DFAT	2.19
SIR4	Bohn et al., 2000	ECB-SDR	5.60
	Papst et al., 2004	ECB-SDR	17.72
	Ordas et al., 2009	MCB-STL	7.20
SIR5	Courtial et al., 2014	Sga_P-CA	12.70
	Krakowsky et al., 2005	ADL	17.00
	Papst et al., 2004	ECB-TL	15.84
	Bohn et al., 2000	ECB-TL	6.60
	Barriere et al., 2008	p-CA	10.90
	Samayoa et al., 2014	MCB-ShR	15.32
	Schön et al., 1993	ECB-TL	15.70
SIR6	Barriere et al., 2008	ADL/NDF	5.90
	Jampatong et al., 2002	ECB-TL	8.10
	Krakowsky et al., 2005	NDF	13.00
	Santiago et al., 2016	p-CA	1.23
SIR7	Schön et al., 1993	ECB-TL	3.90
	Riboulet et al., 2008	ADL/NDF	17.70
	Krakowsky et al., 2005	NDF	23.00
	Cardinal et al., 2003	STNDF	9.90
SIR8	Fontaine et al., 2003	Hcell	12.20
	Barriere et al., 2008	Est FA	16.90
	Roussel et al., 2002	Hcell/NDF	11.30
	Krakowsky et al., 2004	ECB-ST	21.30
	Santiago et al., 2016	p-CA	10.53
SIR9	Roussel et al., 2002	Cell/NDF	10.40
	Cardinal et al., 2001	ECB-TL	11.20
SIR10	Santiago et al., 2016	MCB-TL	2.46
	Krakowsky et al., 2004	ECB-ST	11.00
	Cardinal et al., 2003	STNDF	15.10
	Cardinal et al., 2001	ECB-TL	7.80
	Santiago et al., 2016	DFAT	4.00
SIR11	Barriere et al., 2008	Est FA	11.40
	Courtial et al., 2013	ADL/NDF	17.20
	Courtial et al., 2014	p-CA	15.80
	Schön et al., 1993	ECB-TL	13.50
SIR12	Fontaine et al., 2003	est FA	7.77
	Cardinal et al., 2003	STADL	7.10
	Roussel et al., 2002	NDF	16.50
	Cardinal et al., 2001	ECB-TL	6.80
	Barriere et al., 2008	Va	9.40
	Krakowsky et al., 2005	ADL	6.00
SIR13	Barriere et al., 2008	SHNDF	22.60
	Cardinal et al., 2001	ECB-TL	10.80
	Courtial et al., 2014	Van	17.40
	Krakowsky et al., 2004	ECB-ST	8.10
SIR14	Krakowsky et al., 2002	ECB-ST	24.70
	Krakowsky et al., 2005	ADF	30.00
	Courtial et al., 2013	ADL/NDF	11.80
	Schön et al., 1993	ECB-TL	5.70
SIR15	Santiago et al., 2016	p-CA	1.23
	Roussel et al., 2002	ADL/NDF	10.50
	Fontaine et al., 2003	ADL/NDF	13.42
	Ordas et al., 2010	MCB-STL	9.60
	Courtial et al., 2014	EthFA	25.00
	Barriere et al., 2008	p-CA	6.00
SIR16	Papst et al., 2004	ECB-TL	15.90
	Bohn et al., 2000	ECB-TL	6.30
	Papst et al., 2004	ECB-SDR	12.42
	Fontaine et al., 2003	est p-CA	7.44
SIR17	Roussel et al., 2002	Hcell/NDF	12.90
	Méchin et al., 2001	ADL	7.60
	Krakowsky et al., 2002	ECB-ST	12.60
	Santiago et al., 2016	p-CA	2.67
SIR18	Courtial et al., 2014	Van	15.10
	Krakowsky et al., 2005	NDF	15.00
	Krakowsky et al., 2004	ECB-ST	9.00
SIR19	Krakowsky et al., 2002	ECB-ST	9.00
	Courtial et al., 2014	Sga-p-CA	13.40
	Bohn et al., 2000	ECB-TL	5.40
	Papst et al., 2004	ECB-TL	7.38
SIR20	Bohn et al., 2000	ECB-TL	3.50
	Samayoa et al., 2014	MCB-ShR	20.20
	Krakowsky et al., 2005	ADL	7.00
	Papst et al., 2004	ECB-TL	5.46
SIR21	Jampatong et al., 2002	ECB-TL	14.00
	Roussel et al., 2002	ADL/NDF	13.50
SIR22	Bohn et al., 2000	ECB-SDR	5.70
	Cardinal et al., 2001	ECB-TL	13.80
	Krakowsky et al., 2005	ADF	11.00
	Cardinal et al., 2003	STADF	10.70
	Barriere et al., 2008	5-5 diFA	9.00
SIR23	Courtial et al., 2013	ADL/NDF	13.80
	Krakowsky et al., 2002	ECB-ST	21.30
	Krakowsky et al., 2004	ECB-ST	6.20
	Krakowsky et al., 2005	NDF	16.00
	Jampatong et al., 2002	ECB-TL	15.00
SIR24	Krakowsky et al., 2004	ECB-ST	10.00
	Jampatong et al., 2002	ECB-TL	6.90
	Courtial et al., 2013	ADL/NDF	6.80
	Roussel et al., 2002	ADL/NDF	10.50
SIR25	Courtial et al., 2014	8-O-4diFA	12.40
	Krakowsky et al., 2005	ADF	17.00
	Krakowsky et al., 2004	ECB-ST	7.20
SIR26	Papst et al., 2004	ECB-SDR	17.20
	Courtial et al., 2014	ADL/NDF	42.50
	Courtial et al., 2013	ADL/NDF	37.70
	Santiago et al., 2016	MCB-TL	3.17
SIR27	Courtial et al., 2014	p-CA	9.10
	Papst et al., 2004	ECB-SDR	16.80
	Courtial et al., 2013	KL/NDF	16.30
SIR28	Jampatong et al., 2002	ECB-TL	8.10
	Fontaine et al., 2003	Hcell	24.78
	Santiago et al., 2016	p-CA	5.72
	Roussel et al., 2002	Hcell/NDF	27.70
	Krakowsky et al., 2005	NDF	9.00
SIR29	Krakowsky et al., 2004	ECB-ST	4.70
	Fontaine et al., 2003	KL/NDF	6.79
SIR30	Krakowsky et al., 2004	ECB-ST	10.40
	Papst et al., 2004	ECB-TL	5.50
	Barriere et al., 2008	Va	11.90
	Cardinal et al., 2001	ECB-TL	6.10
	Méchin et al., 2001	CPC	9.30
SIR31	Santiago et al., 2016	p-CA	1.67
	Krakowsky et al., 2005	ADF	21.00
	Schön et al., 1993	ECB-TL	3.70
	Papst et al., 2004	ECB-SDR	8.20
SIR32	Krakowsky et al., 2005	NDF	10.00
	Krakowsky et al., 2004	ECB-ST	6.10
SIR33	Santiago et al., 2016	DFAT	4.72
	Barriere et al., 2008	Va	21.10
	Papst et al., 2004	ECB-SDR	13.34
	Jampatong et al., 2002	ECB-TL	4.10
SIR34	Ordas et al., 2010	MCB-RSTL	15.00
	Barriere et al., 2008	Va	10.00
	Papst et al., 2004	ECB-SDR	20.00
SIR35	Samayoa et al., 2014	MCB-ShR	16.06
	Cardinal et al., 2001	ECB-TL	4.40
	Cardinal et al., 2003	STADL	4.10
SIR36	Fontaine et al., 2003	est p-CA	8.73
	Krakowsky et al., 2004	ECB-ST	5.00
SIR37	Krakowsky et al., 2004	ECB-ST	13.70
	Cardinal et al., 2001	ECB-TL	11.40
SIR38	Fontaine et al., 2003	KL/NDF	7.44
	Cardinal et al., 2003	STADL	0.30
	Courtial et al., 2014	p-CA	15.80
	Courtial et al., 2013	KL/NDF	16.20
	Samayoa et al., 2014	MCB-TL	11.27
	Cardinal et al., 2001	ECB-TL	7.60
	Bohn et al., 2000	ECB-TL	7.40
SIR39	Fontaine et al., 2003	ADL/NDF	10.32
	Roussel et al., 2002	Hcell/NDF	15.80
	Ordas et al., 2009	MCB-STL	10.80
SIR40	Krakowsky et al., 2004	ECB-ST	7.60
	Barriere et al., 2008	Sg	19.10
	Krakowsky et al., 2005	ADF	11.00
	Santiago et al., 2016	DFAT	1.52
	Santiago et al., 2016	FA	2.74
SIR41	Bohn et al., 2000	ECB-TL	8.10
	Barriere et al., 2008	ADL/NDF	16.50
	Schön et al., 1993	ECB-TL	4.90
SIR42	Papst et al., 2004	ECB-TL	5.82
	Krakowsky et al., 2004	ECB-ST	8.80
	Cardinal et al., 2003	STADL	12.90
	Papst et al., 2004	ECB-SDR	10.90

*8,5′-DiFA b, 8,5′ Diferulic acid; ADL, acid detergent fiber; ADL, acide detergent lignin; AP, adult progeny emergence; CFP, p-coumaroyl-feruloyl putrescine; CPC, crude protein content; DFAT, total diferulic acid; DI, Doby index of susceptibility; Est FA, ester ferulic acid; Est p-CA, ester p-coumaric acid; Eth FA, ether ferulic acid; FA, ferulic acid; FP, flour production; Gal, Galactose; GD, grain damage; Glc, glycose; GWL, grain weight loss; Hcell, hemicellulose; HRGP I, hydroxyprolinerich glycoprotein insoluble; KD, kernel damage; KL, klason lignin; KR, kernel resistance; LBD, leaf blade damage; LDR, leaf damage rating; LFD, leaf feeding damage; MCB, Mediterranean corn borer; NDF, neutral detergent fiber; p-CA, p-coumaric acid; RSTL, relative stalk tunnel length; SCB, Sugarcane borer; SDR, stalk damage rating; sga-p-CA, syringaldehyde acylated by p-CA; ShR, shank resistance; ST, stalk tunneling; STL, stalk tunnel length; SWCB, southwestern corn borer; TL, tunnel length; TPhA, total phenolic acid; trans-FA, trans-ferulic acid; Va, vanillin; SH or ST preceding ADL, ADF, ADL, or NDF means the components were measured from the leaf-sheath or stalk tissues, respectively*.

Although in this study more than half of the total populations included in the meta-analysis were of advanced generations (Table 1), most of the MQTL feature a combination of primary and advanced populations. Furthermore, most of the QTL experiments included in the analysis used the line B73 as a parent for their bi-parental populations (Table 1), and as a result, the majority of LIR, SIR, and KIR MQTL contain at least one original QTL identified from a population parented by B73. Also, most of the MQTL identified in this study were representative of temperate, and to some extent, subtropical and tropical maize populations (Tables 1, 4).

We sorted out the different tissue-specific MQTL by their genetic and physical position which revealed 14 regions showing overlaps among MQTL for resistance in different tissues of which, seven involve KIR MQTL, and three combine all the resistance categories (Table 3).

### MQTL co-localization with other insect-related QTL, and genome coverage

We compared the physical positions of the QTL for insect resistance experiments that we failed to consider in the meta-analysis with those of the declared MQTL and located them in several MQTL taking into consideration of the tissues involved (Table 3). We estimated the percentages of genetic coverage of the MQTL for KIR, LIR, and SIR and that of their projected QTL by summed up the 95% CI in each case while correcting for overlaps in the case of the QTLs, and computing their percentages against the genetic size of the reference map following the formula:

$$\begin{array}{l} \left(\frac{QTL/MQTL\;total\;genetic\;coverage}{Reference\;map\;genetic\;size}\right)\;*\;100. \end{array}$$

To compute the percentage of genetic coverage reduction achieved by the meta-analysis, we used the formula:

$$\begin{array}{l} \left(\frac{QTL\;total\;genetic\;coverage\;-\;MQTL\;total\;genetic\;coverage}{QTL\;total\;genetic\;coverage}\right)\;*\;100. \end{array}$$

Thus, the meta-analysis allowed reduction of the genetic coverage from 33.86 to 13.91%, 50.43 to 14.45%, and 69.65 to 13.84%, for KIR, LIR, and SIR, respectively, amounting to a reduction from QTL to MQTL coverage of 58.91, 71.35, and 80.12%, respectively (Table 3). We also estimated the genome coverage of the KIR, LIR, and SIR MQTL by adding up all the differences between the end and start physical positions of the MQTL in each class and computing their percentages against the physical length of the B73 version 2 which is 2,066,432,718 base pairs (bp) (https://genomevolution.org/coge/OrganismView.pl) using the formula:

$$\begin{array}{l} \left(\frac{MQTL\;total\;physical\;coverage}{2,066,432,718}\right){\;}^{*}\;100. \end{array}$$

Thus, the genome coverage for the different types of resistance is 21.25, 17.07, and 25.03%, for KIR, LIR, and SIR, respectively.

## Discussion

Combinatorial insect attacks on leaves, stems, and kernels severely limit maize yield, and QTL identification was intended to serve as a basis for genetic improvement through marker-assisted breeding programs. However, several factors inherent to experimental and statistical procedures limit the efficient use of QTL (Jiang, 2013). Insect resistance being polygenic and controlled mostly by several small effect QTL, an efficient way of making the QTL information useful in molecular breeding is through a meta-analysis (Wang et al., 2016). Several studies reported the correlations between CWC and insect resistance, especially SB and SP (Santiago et al., 2013). However, the accumulation of CWC in maize varies substantially between tissues and even within the same tissue over time. Hence their involvement in insect resistance varies accordingly (Santiago et al., 2013). Co-localizations of QTL for resistance to different insect species prompted the investigation of MQTL involving multiple insect resistance that would assist in breeding programs for multiple resistance to pests. In this study, QTL for maize resistance to SB and SP, and for maize CWC discovered in leaves, stems, and kernels were separately meta-analyzed using the IBM2 2008 Neighbors (www.maizegdb.org) as a reference map to identify significant MQTL for insect resistance in different maize tissues with potential use in multiple pests' resistance molecular breeding.

### QTL meta-analysis is efficient in refining QTL CIS and reducing QTL genome coverage

Although the number of original QTL in each tissue was substantially reduced, the resulting MQTL are relatively large constituting a limitation for their introgression using MAS. Large CIs resulting from a meta-analysis is not seldom for studies conducted in similar conditions (Jin et al., 2015; Luo et al., 2015; Zhao et al., 2015; Jiang et al., 2016). In fact, the IBM 2 2008 neighbors is a result of intermating lines from a bi-parental cross between B73 and Mo17, and as such, has a size increase of nearly four-fold in the genetic map distance, but also it substantially increases resolution up to 91% (Lee et al., 2002). Therefore, map projection of the original QTL on the reference map results in an increase of the CIs for the individual QTL as a result of the homothetic rescaling of the QTL CI (Sosnoswki and Joets, 2012) leading to a similar increase of the CIs of the MQTL. Also, the increase of MQTL CIs is caused by the fact that the 95% CIs of the QTL, considered conservative and more comprehensive of the real span of the individual QTL (Truntzler et al., 2010), are mostly larger than the original CIs due to low QTL *R*^2^. Despite these increases in the size of the CIs, the meta-analysis though BioMercator (Arcade et al., 2004) permitted reduction of the genome size covered by the QTL from 33.86 to 13.91%, 50.43 to 14.45%, and 69.65 to 13.84%, for KIR, LIR, and SIR, respectively. Similar results were obtained by Truntzler et al. (2010) who meta-analyzed maize CWC and digestibility and succeeded to refine QTL CIs and reduce the QTL genome size coverage from 68 to 28%. However, the genome coverage based on the physical coordinates of the MQTL is higher than that based on the genetic map reaching around 17, 21, and 25% of the total maize genome for LIR, KIR, and SIR, respectively. This difference between the genetic and genomic coverage of the MQTL is because the locus pair lookup tool (Andorf et al., 2010) provides a range estimate of physical coordinates of the MQTL's flanking makers which results in wider physical lengths of the MQTL CIs.

### Cell wall constituents within the meta-QTL

Plants co-evolved with insects and developed an array of resistance mechanisms to thwart herbivore attacks through direct or indirect defense mechanisms (War et al., 2012). Consequently, plant-insect interaction is the primary driving force of plants' evolution, especially the development and conservation of a diverse range of defense metabolites and their underlying genes (Kliebenstein, 2014). The results of this study corroborate the significant role played by plant chemicals with the involvement of at least one CWC QTL in the majority of the identified insect resistance MQTL and confirms earlier correlations (Groh et al., 1998; Papst et al., 2004; Cardinal and Lee, 2005; Krakowsky et al., 2007; García-lara et al., 2010; Santiago et al., 2016). Also, several co-localizations between fiber and hydroxycinnamates, and between hydroxycinnamates and sugars occur within the SIR and KIR MQTL, respectively. However, a co-localization between two or more QTL does not necessarily mean they control the same phenotypes since a QTL is a genomic region that can contain several genes that could be playing different functions. In fact, QTL co-localizations result from mainly two reasons. One reason is gene pleiotropism whereby genes under the MQTL regions regulate the production of CWC conferring a protective function against several insects through the fortification of the maize cell wall and antibiosis or antixenosis (Smith and Clement, 2015). It could also be due to tight-linkage of QTL/genes not resolved by the meta-analysis. Ferulates and *p*-coumarates are reported to form several structures through cross-linking and binding with fiber components such as hemicellulose and lignin which in turn act as a barrier to leaf, stem, and kernel feeding by insects (Santiago et al., 2013, 2017), which, in this study could explain the co-localization of QTL for these components in most SIR MQTL. Besides, 14 chromosomal regions contain overlaps between MQTL for resistance in different tissues of which, seven involve KIR MQTL and three combine all the resistance categories suggesting possible common resistance components in these MQTL. Transcription factors that regulate plant secondary metabolism genes are mostly tissue-specific but also can be ubiquitous (Pichersky and Gang, 2000; Vom Endt et al., 2002; LeClere et al., 2007). Furthermore, plants can use defense mechanisms specific to one tissue or condition to respond to stresses in another compartment through the production of new specific enzymes that could be functional variations of existing ones and arise from genes routinely expressed in specific conditions (Pichersky and Gang, 2000). Thus, the overlaps between different tissue-specific MQTL are worth further investigations to test the hypothesis of common resistance mechanisms across tissues.

### MQTL for multiple insect resistance

Despite its importance in trait genetic architecture analysis, QTL meta-analysis for maize resistance to insect-related stresses has been only reported for ear rot rates and mycotoxin contaminations due to *Aspergillus flavus, Fusarium graminearum*, and *F. verticillioides* (Xiang et al., 2010; Mideros et al., 2014). This study is the first report of QTL meta-analyses on maize resistance to insect herbivory and corroborates the polygenic nature of maize resistance to SB and SP (Kliebenstein, 2014). Most LIR and SIR MQTL discovered involve multiple insect resistance confirming correlations among resistance mechanisms to diverse stem borer species (Thome et al., 1992). Some of LIR MQTL like LIR4, 17, and 22 involve resistance to ECB, SCB, and SWCB. Regarding QTL experiments for stem insect resistance, only ECB and MCB were involved. Therefore, multiple insect resistance MQTL were related to these two insect pests, for instance, SIR2-5, 10, 20, 26, 34, 34, and 38. Furthermore, the kernel insect resistance MQTL, KIR3, 15, and KIR18 involve QTL for resistance to kernel damage by MCB and MW and could be good candidates for reducing maize grain damage and mycotoxin contamination attributable to insect pests. These multiple-insect resistance tissue-specific MQTL, when used together in a MAS scheme, could help in sustainably improving maize resistance to a broad range of insect pest species.

The omnipresence of ECB QTL in most of the MQTL identified in this study might be due solely to the fact that more experiments, thus more QTL for ECB were included (Table 1). It is probable that if more experiments were previously conducted on other SB and SP species as required for polygenic traits (Collard et al., 2005; Ordas et al., 2009), the meta-analyses would have generated more valuable MQTL. Thus, a comprehensive review of stem borer resistance-related mapping experiments provided by Meihls et al. (2012) located the different QTL discovered in the maize bins. A comparison between the bin locations of the MQTL from our study with that of the QTL in the experiments we could not include in the meta-analysis (Groh et al., 1998; Brooks et al., 2007; Samayoa et al., 2015a,b; Jiménez-Galindo et al., 2017) shows co-localization in the same or adjacent bins. These co-localizations were further illustrated by locating some of these QTL (Groh et al., 1998; Brooks et al., 2007; Samayoa et al., 2015a,b; Jiménez-Galindo et al., 2017) within the corresponding tissue-specific MQTL based on the physical positions of their flanking markers. However, several other QTL from these studies did not fall within the CIs of the MQTL corroborating the probability of the existence of more tissue-specific MQTL. Furthermore, only KIR18 combines QTL discovered from both of the MW resistance QTL mapping experiments (García-lara et al., 2009; Castro-Álvarez et al., 2015). The lack of QTL co-localizations between these two studies necessitates conducting more QTL experiments on MW resistance on diverse maize background to confirm the discovered QTL and identify additional MW resistance genomic regions (Castro-Álvarez et al., 2015). In this meta-analysis, no QTL experiment conducted in Africa could be included, yet maize resistance to local insects, especially stem borer species could be having a different genetic basis due to the co-evolutionary and environment-dependent nature of plant-insect interactions (War et al., 2012; Kliebenstein, 2014). Therefore, more QTL discovery studies for resistance to local stem borers such as *Busseola fusca* and *Chilo partellus* need to be conducted in addition to the already available ones (Munyiri and Mugo, 2017) to allow more comprehensive comparative mappings to be carried out. These recommendations also hold for other parts of the world such as Central and South-America, and Asia maize germplasms and stem borer and storage pest species.

### Implications for multiple insect resistance breeding

The maize pan-genome theory supports the commonality of a substantial portion of the maize genome, containing almost all the genes, in all lines (Morgante et al., 2007). Maize experiences several simultaneously or subsequently occurring abiotic and biotic stress, and such stress events lead to the generalization of fitness phenotypes across environments among other adaptive strategies (Anderson et al., 2013). A generalization of insect resistance across populations of diverse genetic and geographic origins would allow developing multiple pest resistance by taking advantage of available data. This meta-analysis allowed us to investigate the commonality of the genetic basis maize resistance to insects of geographically diverse environments among both genetically and geographically diverse maize populations. However, QTL studies have mostly been conducted using temperate materials from North America and Europe. The genetic variability in other germplasms such as African and Central and Southern American and Asian materials have been poorly explored, hence, underrepresented in the meta-QTL identified in this study. Therefore, the current MQTL study might not have comprehensively uncovered all possible genomic regions involved in maize resistance to stem borers and storage pests. Nonetheless, we identified several consensus QTL, and as per the pan-genome theory, we can assume that the MQTL identified are inclusive of most of the insect resistance genes (Morgante et al., 2007) contained in European and North American germplasm. Also, most of the MQTL identified in this study are representative of all the geographical diversity and the different recombination levels of the host plant populations used in the original QTL mapping experiments. The diversity in these MQTL implies a commonality of the genomic regions responsible for multiple pest resistance across populations and generations. Conducting QTL mapping studies in other regions of the world and including them in a more comprehensive meta-analysis would help to better understand the extent of this convergence of resistance genomic regions in maize.

Insect resistance QTL in maize have low *R*^2^ due to the influence of low to moderate heritabilities (García-lara et al., 2009), and several QTL are involved with usually small effects and large CI in controlling the trait (Jiang, 2013), especially for maize resistance to SP (García-lara et al., 2009). Low heritabilities and large CIs imply low efficacy in MAS (Ordas et al., 2009). However, in this meta-analysis, the MQTL's CIs and genome coverage are still large, which could be solved by conducting more QTL discovery studies using more precise methods. Nonetheless, the MQTL identified here can help in efficiently achieving multiple pest resistance by accumulating into commercially preferred but susceptible lines through molecular breeding approaches. Furthermore, the overlaps observed among MQTL from different tissues prompts the investigation of combined resistance across maize tissues and insect pest species which is feasible through multi-trait association mapping (Stich et al., 2008) among other methods.

The polygenic nature of combined resistance highlighted in this study implies that the most effective methods for molecular breeding of multiple-insect resistant lines would be marker-assisted gene pyramiding or marker-assisted recurrent selection (Jiang, 2013) and could be combined with phenotypic selection for better breeding progress when dealing with insect resistance with low heritability (Collard and Mackill, 2008). The MQTL identified in the current study cannot be readily utilized in regions, for instance, the sub-Saharan Africa, whose germplasms was limitedly included in the meta-analysis. In a bid to accelerate breeding, regional genome-wide association studies (Chen et al., 2017) could be conducted to investigate resistance trait-related SNPs/INDELs within the MQTL identified in this study as a confirmatory step before use in multiple insect pests' resistance molecular breeding.

## Author contributions

AB conceived and developed the study with the help of PR, SK, LM, MO, TO, and DK. AB run the analyses with inputs from DO, SA, LM, and DK. The manuscript was drafted by AB and was critically reviewed by all the authors with final approval by PR, SK, MO, LM, and TO.

### Conflict of interest statement

The authors declare that the research was conducted in the absence of any commercial or financial relationships that could be construed as a potential conflict of interest. The reviewer AB and handling Editor declared their shared affiliation.
